# Does Personality Modulate the Sensitivity to Contaminants? A Case Study with Cadmium and Caffeine

**DOI:** 10.3390/toxics13030147

**Published:** 2025-02-21

**Authors:** Niedja Santos, Sara Reis, Inês Domingues, Miguel Oliveira

**Affiliations:** Department of Biology & CESAM, University of Aveiro, Campus Universitario de Santiago, 3810-193 Aveiro, Portugal

**Keywords:** stress-coping styles, swimming behavior, short-term effects

## Abstract

Personality has been reported to influence fish response to stress. This study aimed to assess whether shy and bold fish display different sensitivities to two environmental contaminants: caffeine (CAF) and cadmium (Cd). Thus, the sensitivity to Cd was compared based on lethal concentrations (LCs). The potential different response to CAF, known to alter the social behavior and locomotor activity of zebrafish, was studied using behavioral parameters. Overall, different LC values were found for each group: 48 h LC_50_ values of 4.79 (shy fish) and 8.20 mg·L^−1^ (bold fish); and 96 h LC_50_ values of 3.79 (shy fish) and 9.79 mg·L^−1^ (bold fish). In terms of response to CAF, a significant interaction between CAF and personality traits (bold and shy) was found in the locomotion activities (distance travelled, and medium and rapid movements), in the mirror test (frequency of contact and entries into the contact, approach, and distant zones), and in social tests (swimming distance in zones 2 and 3; time spent in zones 1, 2, and 3; and number of entries into zones 1 and 2). Shy fish exposed to 300 μg·L^−1^ of CAF presented hypoactivity, reduced aggressive behavior, and reduced sociability. Conversely, CAF did not influence the behavior of bold fish. In general, shy fish were more sensitive to Cd and exhibited anxious behavior when exposed to CAF, which appears to be the factor responsible for changes in their social behavior. Our results highlight the importance of taking personality traits into account in future studies, as variations in behavioral responses between bold and shy individuals can mask the toxicological effects of different chemicals.

## 1. Introduction

Personality traits are defined by individual behavioral traits that are persistent over time and that are expressed in different situations [1,2,3,4,5]. Cattell and Cattell [6] through empirical observation and statistical analysis, identified and defined 16 personality traits, including the bold and timid (shy) axis. The bold and shy axis stands out as a distinct, hereditary, and stable source of behavioral diversity [7]. Although this concept was initially described to detect individual differences in humans, personality traits are common among animals in the natural world [6,8]. In this regard, based on stable characteristics, including stress response, aggression, sociability, and the ability to explore a new environment, human and non-human individuals can be classified by personality [7,9,10]. Bolder profiles typically represent proactive individuals, dominant in social hierarchies, more aggressive, and more willing to take risks, and are also characterized by lower stress levels and a stronger inclination towards routines compared to their shy counterparts [7,9,10,11]. These inherent individual distinctions give rise to a range of cognitive skills in response to specific challenges, such as food and mate search, competition, and predator avoidance in fish [12]. Overall, bold and shy individuals display distinct susceptibilities to stressors. This divergence results from variations in how these individuals adapt to different environmental conditions, a process influenced by physiological differences (e.g., metabolic rate and neuroendocrine variations) [13,14].

The study of the links between personality traits and responses to various stressors in fish has been attracting the interest of the scientific community given the potential of personality traits to modulate the impacts of stressors on different organisms (e.g., fish) and consequently interfere in risk assessment. It has been reported that the effects of alcohol on shoaling, swimming speed, and exploration are modulated by personality traits [15,16,17,18]. Different responses have also been reported for nicotine (e.g., shy fish exhibited stronger anxiety-type responses than bold fish, as they reduced exploratory behavior [15]) and microplastics (bold zebrafish exhibited higher feeding activity levels, captured microplastics more frequently, and ingested a greater quantity of microplastics compared to shy zebrafish [19]).

Zebrafish have emerged as a good model for studying complex behaviors, including characteristics related to personality traits [15,16,17,18,19,20,21,22,23]. Different studies on fish personality usually assess the exploratory capacity (e.g., novel tank, T-labyrinth test, emergence test, feeding test), sociability (social test and/or shoaling test, mirror test), habituation to light–dark stimulus, and physiology of animals [2,24,25,26]. Nevertheless, there is a shortage of research regarding the shy and bold axis and its impact on responses to chemicals (e.g., pharmaceutical products) detected in the aquatic environment, as well as the resulting ecological consequences.

This study aimed to assess the sensitivity of personality traits (bold versus shy) to two chemicals known as environmental contaminants: cadmium (Cd) and caffeine (CAF). Human exposure to Cd and CAF has been associated with toxic effects. Cd toxicity is largely driven by oxidative stress, leading to DNA damage, impaired cell function, and organ toxicity in the heart, brain, liver, lungs, and kidneys [27]. Additionally, adverse effects of CAF consumption in healthy populations, particularly at high doses (above 400 mg/day), have been linked to cardiovascular, behavioural, reproductive, developmental, bone, and calcium disturbances [28,29].

Cd is a widely studied environmental pollutant and is considered a public health problem due to its widespread presence in various environmental compartments and its ability to cause toxicity in animals even at low concentrations, as well as its ability to bioaccumulate [30,31]. CAF is known as a marker of anthropogenic contamination and responsible for inducing behavioural alterations in zebrafish at concentrations ranging from μg to mg·L^−1^ [32,33,34,35,36]. In this regard, this study determined the median lethal concentration (LC_50_) of Cd for bold and shy fish after 96 h of exposure and evaluated the behavioural responses (locomotion and social behaviour) of bold and shy fish after a 7-day exposure to CAF. We hypothesize that personality modulates the response to different environmental stressors.

## 2. Materials and Methods

### 2.1. Zebrafish Culture

Zebrafish (*D. rerio*) (AB strain) were kept in a ZebTEC (Tecniplast, Buguggiate, Italy) recirculating system at the University of Aveiro’s Biology Department (Aveiro, Portugal). Culture water was generated by reverse osmosis, which was then complemented with salt (Instant Ocean Synthetic Sea Salt, Spectrum Brands). The water temperature was maintained at 27.0 ± 1 °C, conductivity at 800 ± 50 μS/cm, pH at 7.5 ± 0.5, and dissolved oxygen at ≥95% saturation. The photoperiod cycle (light–dark) was 14:10 h. Adult fish were fed once daily with GEMMA Micro 500 artificial feed (Skretting^®^, Algeciras, Spain). Adult zebrafish at 1 year old were chosen for the experimental assays. All procedures involving the use of animals were performed in accordance with European Union guidelines and regulations (e.g., OECD 203) and were approved by the Animal Care and Use Committee of the University of Aveiro, Portugal.

### 2.2. Personality Determination: Emergence Test

The selection of animals’ personality based on an emergence test was conducted following the study performed by Mackenzie et al. [37]. The emergence test has been widely used to characterize bold and shy zebrafish based on their willingness to take risks. In this sense, the test was executed to assess an animal’s tendency to leave a safe area and explore a new, less safe area. Briefly, rectangular glass tanks (40.6 cm × 26 cm × 19.5 cm) were used for acclimatization (acclimatization tanks) and for the test itself (test tanks). The test tank was divided into two sections: a small section (15 × 26 cm) where the fish are initially placed and a big section (25.6 × 26 cm). The two parts were divided by a black wall with a hatch that can be opened and closed and that allows fish to move from the small section to the big one. The day before to separation procedure, a set of 45 fish were transferred for a period of 12 h to acclimatization tanks to encourage them to explore the environment [38]. After acclimatization, 9 individuals were randomly selected and introduced into the small compartment of the test tank, with the hatch closed, for 10 min (Step 1). After this period, the hatch was open, allowing the fish to swim freely and explore the other compartment (Step 2). The first 3 fish to cross within 10 min were considered bold [37] and were immediately removed from the test tank and placed in a new tank. Steps 1 and 2 were repeated with the remaining fish, and the 3 individuals passing to the new compartment were labelled as intermediate (and were removed from the experiment). Those that stayed in the small compartment were subjected to Steps 1 and 2 again, but Step 2 was extended to 30 min. Fish that did not move to the other division were classified as shy, while those that did were discarded.

### 2.3. Chemicals

Cadmium chloride hemi(pentahydrate) (CAS number 7790-78-5; 99% purity) was obtained from Sigma Aldrich (Madrid, Spain) and was used as a source of cadmium (Cd).

Analytical grade CAF (1,3,7-Trimethylxanthine; CAS number 58-08-2; 98% purity) was obtained from TCI Chemicals (Zwijndrecht, Belgium).

The stock solution (Cd—250 mg·L^−1^; CAF—10 mg·L^−1^) and test solutions were prepared in the zebrafish water system (Section 2.1).

### 2.4. Sensitivity of Bold and Shy Fish to Cd

In order to determine the median lethal concentration (LC_50_), the zebrafish previously selected as bold or shy were exposed for 96 h to 6 concentrations of Cd (0, 1.80, 3.00, 4.81, 7.70, and 12.31 mg·L^−1^) selected based on preliminary range-finding tests. On the day of exposure, bold (*n* = 60) and shy (*n* = 60) male fish were distributed into 12 tanks (bold = 6 tanks; shy = 6 tanks); each tank contained 10 fish (shy or bold) and 1 L of test solution (0, 1.80, 3.00, 4.81, 7.70, or 12.31 mg·L^−1^). The assay was based on the OECD testing guideline 203 on the Fish Acute Toxicity Test [39]. The temperature, the photoperiod, and the physical–chemical parameters of the water were kept equal to those of the cultivation (Section 2.1). During the test period, the medium was not renewed, and fish were not fed according to OECD 203 [39]. Mortality was recorded every 24 h up to 96 h, to determine LC_50_ values for bold and shy fish.

### 2.5. Exposure to CAF

Bold and shy fish (*n* = 12; ratio of 3 males to 1 female) were exposed to 3 concentrations of CAF (0, 1.5, and 300 μg·L^−1^) in semi-static settings over 7 days. The experiment was conducted in 20 L tank containing 5 L of the test solution and 12 fish per concentration. The concentrations tested were selected based on previous studies that reported the presence of these concentrations in the aquatic environment (e.g., 1.5 μg·L^−1^) [40,41] and their ability to induce changes in exploratory and social behavior of zebrafish exposed after 7 days of exposure (to 0.5. 1.5, and 300 μg·L^−1^) [36]. The medium was renewed every 2 days [42]. The temperature, the photoperiod, and the physical–chemical parameters of the water remained similar to the cultivation conditions (Section 2.1). During the experiment, the fish were fed once a day with the artificial diet Gemma Micro 500 (Skretting^®^, Algeciras, Spain) corresponding to 2% of the weight of the fish in each tank.

### 2.6. Behavior Assays

**Figure 1:**
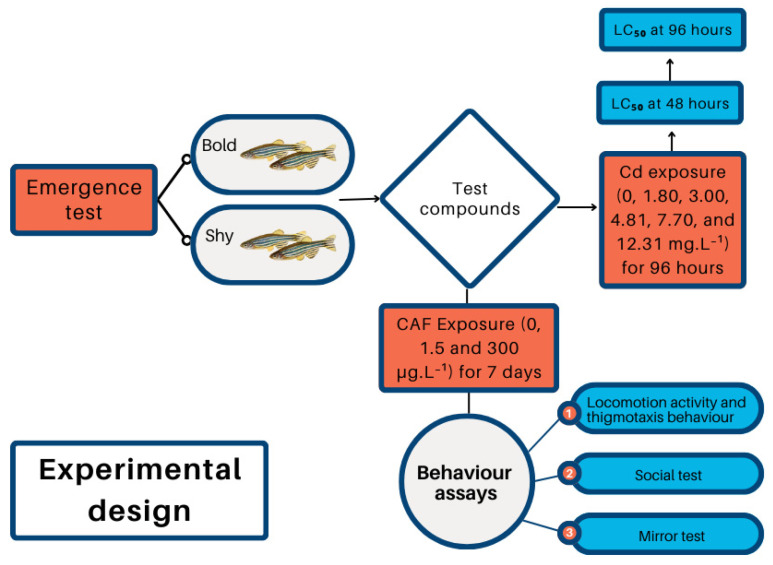
The flowchart illustrates the experimental process developed in this study. The first step involved conducting an emergence test to categorize the fish based on personality traits (bold and shy). The second step consisted of exposing the fish (bold and shy) to two chemicals: (1) Cd, to determine the median lethal concentration (LC_50_) at 48 and 96 h of exposure; and (2) CAF, to assess behavioral changes. Behavioral tests were conducted in the following sequence: locomotor activity and thigmotaxis behavior, followed by the social test, and finally the mirror test.

All fish from each concentration were subjected to the different behavioral tests. Figure 1 illustrates the experimental design and the sequence in which the tests were conducted.

#### 2.6.1. Locomotor Activity and Thigmotaxis Behavior

Locomotion activity and thigmotaxis behavior were performed to assess anxiety-like behavior [43]. The fish were relocated individually into a new aquarium (9.4 cm wide and 14.1 cm long), and locomotor activity and thigmotaxis behavior were recorded using the ZebraBox™ (Viewpoint, Lyon, France) video tracking system. The test started with an acclimatization period (2 min) in the dark, followed by 2 min of light. The following parameters were examined: total distance (mm), swimming time (minutes), swimming speed in each area (slow—less than 8 mm/s; medium—between 8 and 40 mm/s; and rapid—greater than 40 mm/s), and fish path angles (class 1—angles from 180° to 90° and −180° to −90°; class 2—angles from −30° to −90° and from 30° to 90°; class 3—angles from −30° to −10° and 10° to 30°; and class 4—angles from −10° to 0° and 0° to 10°). To assess the fish’s tendency to swim around the edges of the tank, (thigmotaxis behavior), the tank was virtually divided into two zones: an inner zone and an outer zone [43,44].

#### 2.6.2. Mirror Biting Test

To study zebrafish aggressive behavior or sociability, a mirror image stimulation was used [45], following the protocol described by Santos et al. [36]. After experimental exposure, each fish was individually transferred to a rectangular tank (9 cm wide and 14 cm long) containing a mirror on one side. Fish behavior was recorded using the ZebraBox™ (Viewpoint, Lyon, France) video tracking system for 6 min, at 25 frames per second. The protocol consisted of an acclimatization period—1 min of darkness, followed by 5 min of light. For the behavior analysis, the tank was virtually divided in 3 zones: zone 1—zone of contact with the mirror (0.5 cm); zone 2—zone of approach (2.5 cm); and zone 3—zone far from the mirror (10 cm) [46]. The following parameters were analyzed: number of entries per zone, time spent in each zone, and swimming distance in each zone. The time spent in zone 1 was considered as the contact duration between the fish and the mirror. The videos recorded during the test were later analyzed for manual counting of the number of mirror contacts.

#### 2.6.3. Social Test

Zebrafish are social animals that naturally form schools [47]. However, exposure to some chemicals modulates this behavior, increasing (e.g., 17-ethyl estradiol or carbaryl) or decreasing (e.g., CAF) school preference [36,43,48]. The methodology used in this study was adapted from the original protocol by Calcagno et al. [49], for the ZebraBox™ (Viewpoint, Lyon, France) video tracking system. The apparatus consisted of a tank (14 cm long × 6 cm high × 9 cm wide) with two compartments of different sizes (small, 6 cm long × 6 cm high × 9 cm wide; large, 8 cm long × 6 cm high × 9 cm wide) divided by a transparent barrier. The smaller compartment of the tank was used to house three medium-sized adult zebrafish that represented a school of zebrafish. These were placed in the tank before the test began. At the beginning of the experiment, one fish was introduced in the tank’s largest compartment and then its behavior was recorded using the ZebraBox™ (Viewpoint, Lyon, France) video tracking system, for 6 min, at 25 frames per second. The protocol consisted of acclimatization period—1 min of darkness, followed by 5 min of light. For the analysis, the large compartment was virtually divided into three zones of equal size (approximately 2.6 cm): zone 1—zone close to the school; zone 2—intermediate zone; and zone 3—zone far from the school. Then, the swimming distance, swimming time, and number of entries into each zone were analyzed.

### 2.7. Statistical Analysis

Statistical analysis in this study was performed using SigmaPlot V.12.5 (SysStat Inc. software, Chicago, IL, USA). The medium lethal concentration (LC_50_) of Cd was calculated using a 4-parameter logistic model (SigmaPlot 12.5 statistical package). The model choice was decided based on the R2 and the estimated residual standard error.

Data from locomotion, mirror, and social testing were analyzed using a one-way ANOVA (or Kruskal–Wallis test for non-normal distribution data), followed by multiple comparison testing. A two-way ANOVA (personality traits vs. concentration as factors) followed by a Holm–Sidak post-hoc test was performed to study the interaction between personality traits (bold and shy) and CAF. The level of significance for all statistical analyses was 0.05.

## 3. Results

### 3.1. Effects of Cd on Bold and Shy Zebrafish

**Figure 2:**
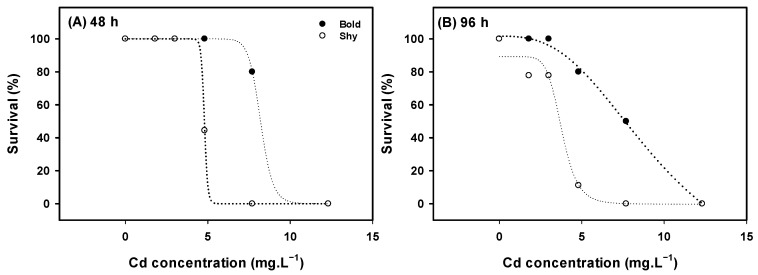
Survival curves of bold and shy zebrafish after exposure to cadmium (Cd) (*n* = 10). (**A**) Survival curve of bold and shy fish after exposure to Cd for 48 h, (**B**) survival curve of bold and shy fish after exposure to Cd for 96 h.

The effects of Cd on bold and shy zebrafish survival were studied after 48 and 96 h of exposure (Figure 2). All shy zebrafish exposed to 7.70 mg·L^−1^ were dead after 24 h, whereas in bold zebrafish total mortality at 24 h was only found for 12.31 mg·L^−1^. The 48 h Cd LC_50_ for bold fish was 8.20 ± 0.035 mg·L^−1^, whereas for shy fish it was 4.79 ± 0.001 mg·L^−1^ (Figure 2A). After 96 h, the estimated LC_50_ of bold fish was 9.79 ± 2.372 mg·L^−1^, whereas for shy fish it was 3.79 ± 0.0447 mg·L^−1^ (Figure 2B).

### 3.2. Effects of CAF on Bold and Shy Zebrafish

#### 3.2.1. Locomotor Activity and Thigmotactic in Zebrafish

**Figure 3:**
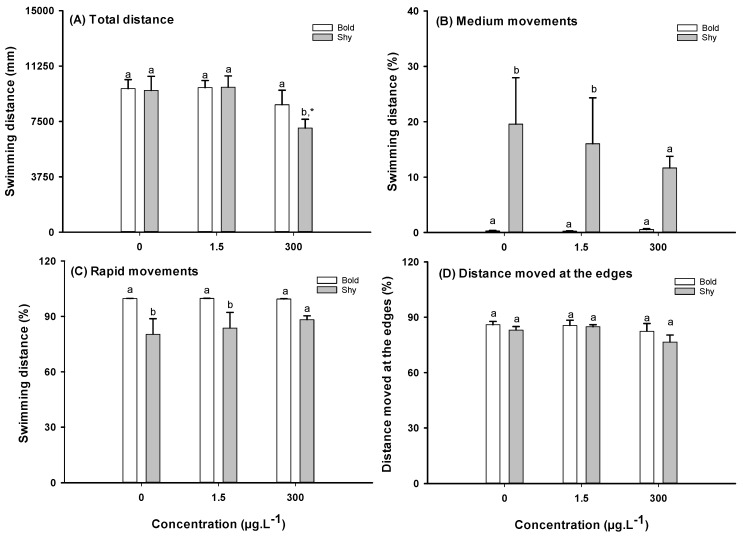
Effects on locomotion activity in bold and shy fish after 7 days of exposure to caffeine (CAF) (*n* = 12). (**A**) Total swimming distance; (**B**) distance travelled in medium movements, and (**C**) distance travelled in rapid movements; (**D**) percentage of distance moved at the edges of the tank by bold (white bars) and shy (grey bar) zebrafish. Data are presented as means ± standard errors. Different letters in bars indicate significant differences between bold and shy fish (*p* < 0.05). Asterisks (*) indicate differences from the respective control (*p* < 0.05).

No significant differences between control bold and shy fish were found in terms of the distance travelled (Figure 3A), the percentage of distance travelled in slow movements (Figure S1, Supplementary Materials), or the distance moved in the edges of the tank (Figure 3D). However, control shy fish swam a significantly greater distance in medium-speed movements than the control bold fish (Figure 3B), whereas control bold fish swam greater distances in rapid movements (Figure 3C). No differences in swimming angles were found between the bold and shy control fish (Figure S1, Supplementary Materials).

Exposure to CAF did not induce significant effects on the locomotion of bold fish. However, shy fish upon exposure to the highest CAF concentration (300 μg·L^−1^) reduced the total distance travelled (ANOVA on ranks (Kruskall–Wallis) followed the Dunn’s post-hoc test (H = 7.650, *p =* 0.022) (Figure 3A).

A two-way ANOVA followed by a Holm–Sidak post-hoc test revealed a significant interaction between CAF and personality traits in the distance travelled (*p* = 0.039), with shy fish decreasing their swimming activity at 300 μg·L^−1^. Bold fish showed no alterations in this parameter. A significant interaction between CAF and personality traits was also found in medium (F_(1,59)_ = 14.861, *p* < 0.001) and rapid movements (F_(1,59)_ = 14.861, *p <* 0.001), with shy fish exposed to 1.5 μg·L^−1^ CAF exhibiting an increase in the percentage of medium movements and a decrease in rapid movements while bold fish did not show alterations in these movements.

#### 3.2.2. Mirror Biting Test

**Figure 4:**
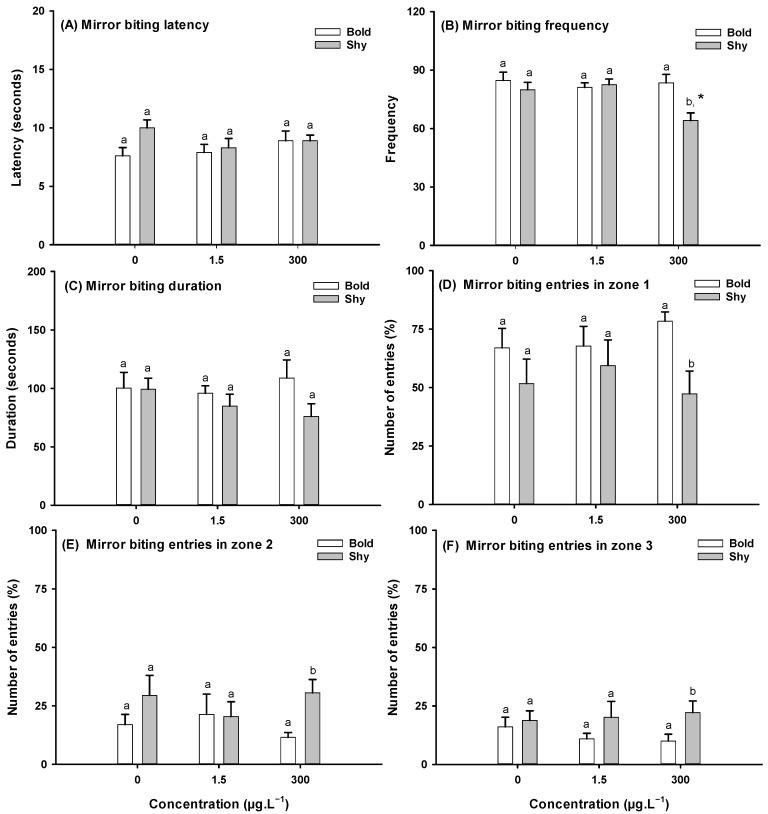
Effects on aggressive behavior in bold and shy fish after 7 days of exposure to caffeine (CAF) (*n* = 12). (**A**) Latency for the first contact with the mirror; (**B**) frequency of contact; (**C**) duration of contact; (**D**) number of entries into zone 1 (contact zone); (**E**) number of entries into zone 2 (approach zone); (**F**) number of entries into zone 3 (zone far from the mirror). Data are presented as means ± standard errors. Different letters in bars indicate significant differences between bold and shy fish. Asterisks (*) indicate differences from the respective control (*p* < 0.05).

No significant differences between bold and shy control fish were found in terms of the latency time to the first mirror approximation (Figure 4A), frequency of mirror bites (Figure 4B), or contact duration (Figure 4C).

Exposure to CAF significantly reduced the frequency of mirror biting in shy fish exposed to 300 μg·L^−1^ (one-way ANOVA followed by Dunnett’s post-hoc test, F_(2,53)_ = 0.05, *p* = 0.012) (Figure 4B) when compared to shy fish in the control group.

The influence of personality on the mirror biting test on the CAF was evaluated by performing a two-way ANOVA followed by a Holm–Sidak post-hoc test. A significant interaction between personality and CAF was observed in the frequency of mirror contact (F_(1,53)_ = 14.861, *p* = 0.001), with shy fish showing a reduction in the frequency of contact with the mirror while bold fish did not exhibit any changes to this parameter. A significant interaction was also observed in the number of entries into zones 1 (F_(1,54)_ = 6.222, *p* = 0.016), 2 (F_(1,54)_ = 6.849, *p* = 0.011), and 3 (F_(1,53)_ = 5.754, *p* = 0.020) of the tanks, with shy fish showing a decrease in the number of entries into zone 1 and an increase in the number of entries into zones 2 and 3 of the tank, while no changes were observed in bold fish.

#### 3.2.3. Social Test

**Figure 5:**
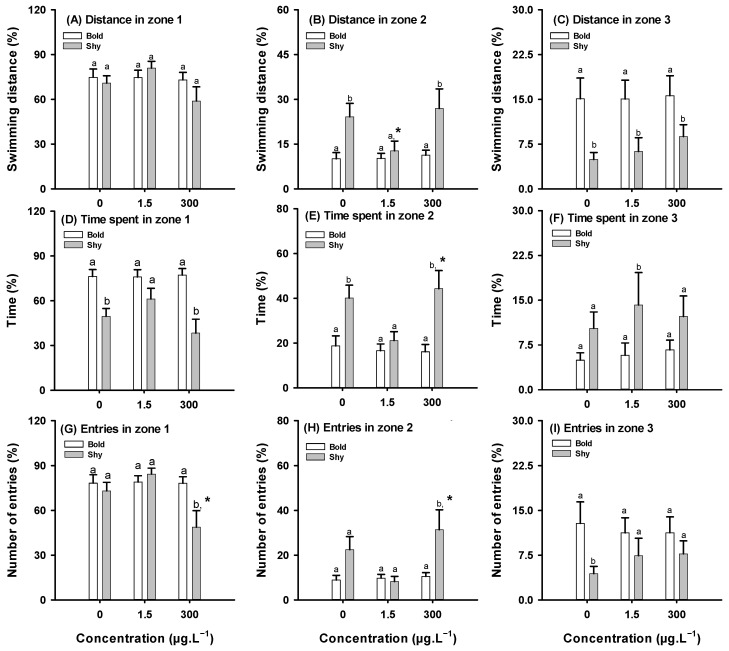
Effects on social behavior in bold and shy fish after 7 days of exposure to caffeine (CAF) (*n* = 12). (**A**) Swimming distance in zone 1; (**B**) swimming distance in zone 2; (**C**) swimming distance in zone 3; (**D**) time spent in zone 1; (**E**) time spent in zone 2; (**F**) time spent in zone 3; (**G**) number of entries into zone 1; (**H**) number of entries into zone 2; (**I**) number of entries into zone 3. Data are presented as means ± standard errors. Different letters in bars indicate significant differences between bold and shy fish (*p* < 0.05). Asterisks (*) indicate differences from the respective control (*p* < 0.05).

The social test revealed that bold and shy fish naturally exhibit distinct social behavior. According to a two-way ANOVA followed by a Holm–Sidak post-hoc test, bold control fish travelled a significantly greater distance in zone 3 (F_(1,58)_ = 14.512, *p* < 0.012) and spent more time in zone 1 (F_(1,59)_ = 27.841, *p* = 0.004) than shy control fish (Figure 5C,D). Shy control fish, on the other hand, spent significantly more time in zone 2 (F_(1,57)_ = 14.384, *p* = 0.006) (Figure 5E). Bold control fish also entered zone 3 (F_(1,59)_ = 5.878, *p* = 0.031) more frequently than shy control fish (Figure 5I).

Exposure to CAF did not elicit significant effects on the social behavior of bold fish. However, shy fish increased or reduced social behaviors compared to the shy control depending on CAF concentration. CAF-exposed shy fish displayed significantly different behavior than their control organisms in a variety of parameters (e.g., distance travelled in zone 2, time spent in zone 2, and number of entries into zones 1 and 2). In terms of distance travelled (Figure 5B) and time spent in zone 2 (Figure 5E), fish exposed to CAF significantly lowered the distance travelled (1.5 μg·L^−1^) (F_(2,57)_ = 2.622, *p* = 0.048) and increased the time spent (300 μg·L^−1^) (F_(2,57)_ = 3.809, *p* < 0.001) when compared to the shy control group. Additionally, shy fish exposed to 300 μg·L^−1^ CAF significantly decreased the number of entrances into zone 1 (F_(2,57)_ = 4.357, *p =* 0.019), and increased in zone 2 (F_(2,57)_ = 6.056, *p <* 0.001) of the tank, when compared to the shy control group.

According to a two-way ANOVA followed by a Holm–Sidak post-hoc test, there was no interaction between the CAF and personality traits in terms of the distance travelled in zone 1 (Figure 5A) or the number of entries into zone 3 (Figure 5I). Interaction between CAF and personality traits was observed in the distance travelled in zone 2 (F_(1,57)_ = 14.384, *p* < 0.001) (Figure 5C) and zone 3 (F_(1,57)_ = 14.512, *p* < 0.001) (Figure 5B) of the tank, with shy fish exposed to CAF showing an increase in the distance travelled in zone 2 and in zone 3, while bold fish did not exhibit changes in the distance travelled in these two areas. A significant interaction was observed in the time travelled in zones 1 (F_(1,59)_ = 27.841, *p* < 0.001) (Figure 5D), 2 (F_(1,59)_ = 20.863, *p* < 0.001) (Figure 5F), and 3 (F_(1,59)_ = 6.682, *p* = 0.012) (Figure 5E) of the tank, with shy fish showing a decrease in time spent in zone 1 and an increase in time spent in zone 2 and zone 3, while bold fish did not show any change in the time spent in any of these areas (Figure 5D–F). A significant interaction was also observed in the number of entries into zone 1 (F_(1,59)_ = 3.565, *p =* 0.002) (Figure 5G) and 2 (F_(1,59)_ = 12.129, *p* < 0.001) (Figure 5H) of the tank, with shy fish showing a decrease in the number of entries into zone 1, and an increase in the number of entries into zone 2, while fish bold did not significantly change the number of entries into different areas of the tank (Figure 5G–I).

## 4. Discussion

This study analyzed the sensitivity of personality traits (bold and shy) to cadmium (Cd) and caffeine (CAF). In the case of Cd, clear differences in Cd sensitivity between bold and shy fish were observed. At 48 and 96 h of exposure, bold fish exhibited greater resistance to Cd as evidenced by a higher LC_50_ value compared to shy fish (Figure 2A,B). Cd and related compounds are environmental pollutants, known for their capacity to cause toxicity in animals and humans, as well as for their ability to bioaccumulate [30,31,50]. LC_50_ values for Cd were previously determined in zebrafish (disregarding personality traits). The estimated LC_50_ values for the same periods tested in the present study were 12.88 mg·L^−1^ at 48 h and 9.68 mg·L^−1^ at 96 h [31]. In the present study, the LC_50_ values of Cd for bold fish were 1.7 and 2.6 times higher than those for shy fish at 48 and 96 h, respectively. One of the pathways by which Cd exerts toxicity in fish is through the increased synthesis of reactive oxygen species (ROS) [51]. It has been observed that shy individuals exhibit higher levels of ROS and subsequent oxidative stress due to elevated stress hormone levels when compared to bold fish [52]. In this context, higher basal ROS levels in shy fish add to the ROS induced by Cd exposure, leading to the higher susceptibility of shy fish towards the compound. In this sense, differences in personality traits may serve as predictors of susceptibility to compounds and physical fitness [53]. However, further studies addressing ROS levels and oxidative stress enzymes activities in both bold and shy zebrafish exposed to Cd are needed for a clearer understanding.

Previous studies revealed that bold fish are more active than shy fish, explore new environments more, spend more time in unprotected areas, and have a greater speed while moving [16,20,54,55,56]. Our data (distance travelled by the control in slow movements, medium movements, and rapid movements) are in agreement with those studies. In terms of locomotion activity (total distance travelled; distance travelled in medium and rapid movements), an interaction between CAF and personality traits could be observed. For example, exposure to 1.5 μg·L^−1^ did not change the distance travelled by shy fish in slow, medium, and rapid movements when compared to the shy control fish. However, they displayed a higher percentage of medium movements and a lower percentage of rapid movements when compared to bold fish subjected to the same conditions (Figure 3B,C). Shy fish exposed to 300 μg·L^−1^ CAF presented hypoactivity (reduced distance travelled), while bold fish showed no differences in these parameters. Hypoactivity associated with increased anxiety levels after exposure to CAF is a behavior commonly described in both larvae and adult zebrafish [33,57]. Santos et al. [36] identified that a 7-day exposure of adult zebrafish to 300 μg·L^−1^ of CAF did not have a significant impact on their locomotion behavior (e.g., distance travelled), although it reduced the vertical exploratory behavior of these fish. In the locomotion test performed in the present study, CAF interacted differently with bold and shy fish. Broadly, bold fish did not display behavioral change when exposed to CAF compared to the control group, which suggests the low susceptibility of these fish to CAF. Exposed shy fish showed significant behavioral variations when compared to shy controls, indicating higher susceptibility to CAF. Factors like variations in metabolic rates between bold and shy fish or the presence of a greater number of type A_1_ adenosine receptors, which are linked to decreased zebrafish swimming activity following CAF exposure, may account for differences in their locomotor activity [58,59]. Differences in bold and shy responses appear to vary depending on the chemical they are exposed to, e.g., in agreement with the effects of CAF observed in this study, such as the increased susceptibility of shy fish. Araujo-Silva et al. [15] reported that shy zebrafish displayed anxiety-like responses following a 60 min exposure to 1 mg·L^−1^ of nicotine when compared to bold fish. On the other hand, bold zebrafish are more susceptible to alcohol than shy fish, as they exhibit anxiety-like behavior when exposed to 0.1% alcohol for 60 min [15].

Bold and shy brook trout (*Salvelinus fontinalis*) show different levels of aggression [56]. In this study, short-term CAF (300 μg·L^−1^) exposure significantly reduced aggressive behavior (reduced the frequency of contact with the mirror and the number of entries into zone 1) or caused shy fish to become more cautious but did not change bold fish behavior. Exposure to CAF is linked to heightened aggression and anxiety in zebrafish. Zebrafish anxious behavior is linked to a decrease in exploratory behavior [33,36,57] and risk-taking. As demonstrated by the locomotion test (e.g., total distance) and the reduced mirror approach, shy fish exposed to 300 μg·L^−1^ of CAF show anxious behavior that is not observed in bold fish. In the mirror test, the shy responses to the mirror image stimulus are in accordance with what has previously been described, i.e., in the presence of acute stress, shy fish tend to respond passively, seeking to “hide/freeze” [60]. On the other hand, it was expected that bold fish would respond actively through “fight/flight” behavior [60]; however, fight behavior was not detected, which may suggest that the CAF concentrations tested were insufficient to alter the behavioral pattern in bold fish. A study carried out by Santos et al. [36] demonstrated that exposure to 0.5, 1.5, and 300 μg·L^−1^ of CAF for 7 days induced aggressive behavior in zebrafish (in undifferentiated fish). On the other hand, similar to the results observed in the present study with shy fish in the mirror test, Gutiérrez et al. [61] reported that a 30 min exposure to 19.4 μg·L^−1^ of CAF led to a reduction in mirror aggression in undifferentiated juvenile zebrafish. Differences in responses between studies (e.g., [36,61]) can be explained by the presence of personality traits. For example, in this study, bold fish exposed to CAF did not change their response to the mirror image; however, shy fish have reduced aggressive behavior. In general, anxiety influences social behaviors. In rats, anxiety-like behaviors encompass social avoidance [62]. The results from the social test performed in this study also support the hypothesis of increased anxiety observed in shy individuals exposed to 300 μg·L^−1^ of CAF, as suggested by locomotion activity and the mirror test results. Exposure to 300 μg·L^−1^ of CAF significantly reduced the sociability of shy fish. In zebrafish, a decrease in social interaction has also been linked to increased anxiety [63]. A study carried out by Santos et al. [36] showed that the exposure of undifferentiated zebrafish to CAF for 7 days reduced sociability at CAF concentrations of 0.5 and 1.5 μg·L^−1^. However, it did not affect fish social behavior when exposed to 300 μg·L^−1^ of CAF. Results similar to those found in this study on social behavior have been described by Araujo-Silva [17], who described that the exposure of shy fish to 0.1% of alcohol for 60 min reduced the sociability of fish. In this regard, the results observed in the present study in both shy and bold zebrafish during the social test agree with the results of the locomotion activity and the mirror test, confirming the difference in the consistent behavioral pattern between these different personality traits.

In general, it was observed that, in this study, the CAF concentrations tested were not able to alter the behavior of bold fish but induced behavioral changes in shy fish. It is known that individual fish behaviors are controlled by endogenous and exogenous factors [60]. Therefore, individual characteristics can explain the differences between the results described by Santos et al. [36] and the results of the present study. For example, the increase in anxiety levels associated with CAF consumption is linked to its interaction with A_1_-like receptors [58]. The exposure of zebrafish to CAF from 1 h post fertilization can increase A_1_ expression [64]. Therefore, variation in the gene expression of these receptors between bold and shy zebrafish could influence the anxiety level displayed by these fish when exposed to CAF. Furthermore, there is evidence that dopamine plays an important role in regulating anxiety. Thörnqvist et al. [55] found that bold zebrafish, characterized by higher risk-taking behavior and increased locomotor activity, exhibit elevated expression of D_2_-type dopamine receptors (drd2a and drd2b) in comparison to shy fish. In general, there are several physiological variations between bold and shy fish, such as changes in metabolic rate and the generation of ROS, which can influence the rate of metabolization and chemical excretion [52,60,65]. In this sense, more studies are needed to understand the differences in CAF metabolism rates and the mechanisms involved in the responses of bold and shy individuals when exposed to chemicals.

## 5. Conclusions

This study evaluated the sensitivity of bold and shy zebrafish when exposed to cadmium (Cd) or caffeine (CAF), two well-studied environmental contaminants. There are differences in Cd sensitivity between bold and shy fish, with bold zebrafish being more resistant to Cd. Shy fish are also more sensitive to CAF. CAF induced anxious behavior in shy fish, detected by reduced locomotion activity, reduced aggressive behavior, and sociability, but had no effect on bold fish. Overall, our findings highlight those differences between shy and bold individuals can influence fish responses during chemical exposure. In conventional tests, where fish are not differentiated based on personality traits, the response to chemicals can vary depending on the proportion of bold and shy fish. The lack of classification of fish by personality traits may mask behavioral effects after chemical exposure. Studying personality traits may reduce the variability in behavioral responses described in the literature in chemically exposed fish.

## Data Availability

The original data presented in the study are openly available in the Supplementary Materials.

## Supplementary Materials

The following supporting information can be downloaded at: https://www.mdpi.com/article/10.3390/toxics13030147/s1, Figure S1: Behavioural effects on bold and shy fish after 7 days of exposure to Caffeine (CAF) (*n* = 12).

## Abbreviations

The following abbreviations are used in this manuscript:

ANOVA	Analysis of variance
Cd	Cadmium
CAF	Caffeine
LC	Lethal concentration
LC_50_	Median lethal concentration
OECD	The Organization for Economic Cooperation and Development
ROS	Reactive oxygen species
