# Supplementary material for: Does Personality Modulate the Sensitivity to Contaminants? A Case Study with Cadmium and Caffeine
Source: Toxics. 2025 Feb 21;13(3):147. doi: 10.3390/toxics13030147 (PMC11946569; doi:10.3390/toxics13030147)
Supplement: Supplementary file 1 [file toxics-13-00147-s001.zip › toxics-3478232-supplementary.pdf]

<u>**Supplementary material**</u>

Does personality modulate the sensitivity to contaminants: a case study with cadmium and caffeine?

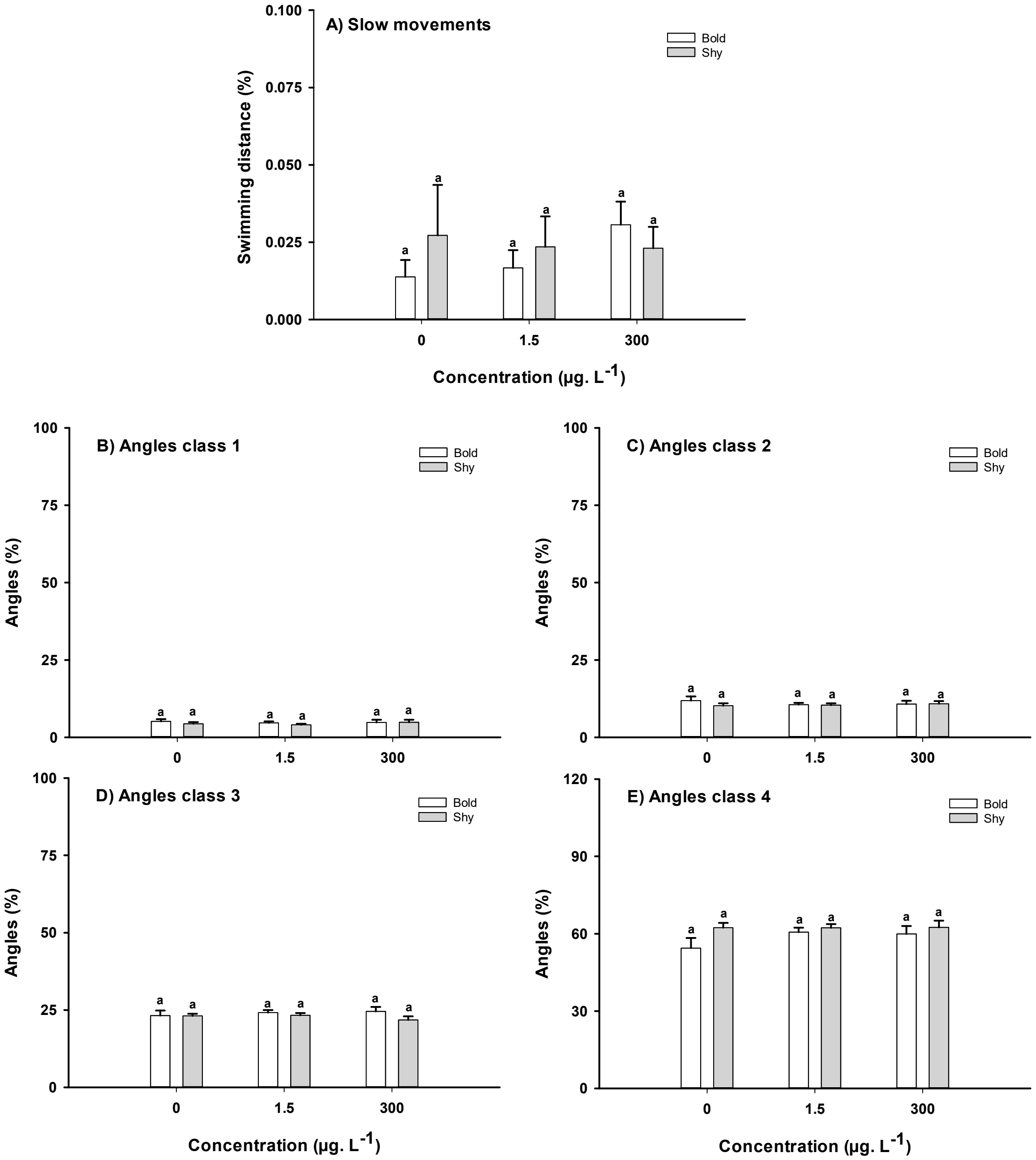

Figure S1. Behavioural effects on bold and shy fish after 7 days of exposure to Caffeine (CAF) (n=12). A) Distance travelled at slow movements; (B) percentage of angles in class 1; (C) percentage of angles in class 2; (D) percentage of angles in class 3; and (E) percentage of angles in class 4. Bold fish are represented by white bars, and shy fish by

grey bars across all graphics. Data are presented as means ± standard errors. Different letters in bars indicate significant differences between bold and shy ($p<0.05$). Asterisks (*) indicate differences to the respective control ($p<0.05$).
